# Chitooligosaccharide prevents vascular endothelial cell apoptosis by attenuation of endoplasmic reticulum stress via suppression of oxidative stress through Nrf2-SOD1 up-regulation

**DOI:** 10.1080/13880209.2022.2133150

**Published:** 2022-10-27

**Authors:** Zin Zin Ei, Pilaiwanwadee Hutamekalin, Peerada Prommeenate, Avtar Singh, Soottawat Benjakul, Kittichate Visuttijai, Pithi Chanvorachote

**Affiliations:** a Department of Pharmacology and Physiology, Faculty of Pharmaceutical Sciences, Chulalongkorn University, Bangkok, Thailand; b Division of Health and Applied Sciences, Faculty of Science, Prince of Songkla University, Songkhla, Thailand; c Biochemical Engineering and Systems Biology Research Group, National Center for Genetic Engineering and Biotechnology, National Science and Technology Development Agency at King Mongkut’s University of Technology Thonburi, Bangkok, Thailand; d International Center of Excellence in Seafood Science and Innovation (ICE-SSI), Faculty of Agro-Industry, Prince of Songkla University, Songkhla, Thailand; e Department of Laboratory Medicine, Institute of Biomedicine, University of Gothenburg, Gothenburg, Sweden; f Center of Excellence in Cancer Cell and Molecular Biology, Faculty of Pharmaceutical Sciences, Chulalongkorn University, Bangkok, Thailand

**Keywords:** ROS, CHOP, ER stress, antioxidant, shrimp shell, chitosan

## Abstract

**Context:**

Endoplasmic reticulum (ER) stress contributes to endothelium pathological conditions. Chitooligosaccharides (COS) have health benefits, but their effect on endothelial cells is unknown. We demonstrate for the first time a protective effect of COS against ER-induced endothelial cell damage.

**Objective:**

To evaluate the protective effect of COS on ER stress-induced apoptosis in endothelial cells.

**Material and methods:**

Endothelial (EA.hy926) cells were pre-treated with COS (250 or 500 μg/mL) for 24 h, and then treated with 0.16 μg/mL of Tg for 24 h and compared to the untreated control. Apoptosis and necrosis were detected by Annexin V-FITC/propidium iodide co-staining. Reactive oxygen species (ROS) were measured with the DCFH_2_-DA and DHE probes. The protective pathway and ER stress markers were evaluated by reverse transcription-polymerase chain reaction, western blot, and immunofluorescence analyses.

**Results:**

COS attenuated ER stress-induced cell death. The viability of EA.hy926 cells treated with Tg alone was 44.97 ± 1% but the COS pre-treatment increased cells viability to 74.74 ± 3.95% in the 250 μg/mL COS and 75.34 ± 2.4% in the 500 μg/mL COS treatments. Tg induced ER stress and ROS, which were associated with ER stress-mediated death. Interestingly, COS reduced ROS by upregulating nuclear factor-E2-related factor 2 (Nrf2), and the oxidative enzymes, superoxide dismutase1 (SOD1) and catalase. COS also suppressed up-regulation of the ER-related apoptosis protein, CHOP induced by Tg.

**Conclusions:**

COS protected against ER stress-induced apoptosis in endothelial cells by suppressing ROS and up-regulation Nrf2 and SOD1. These findings support the use of COS to protect endothelial cells.

## Introduction

Oxidative stress is the imbalance between the generation of reactive oxygen species (ROS) and the antioxidant defense mechanisms of cells and is one of the underlying factors in the pathogenesis and progression of diseases. An excess level of ROS in endothelial cells causes endothelial dysfunction, leading to vascular damage. In addition, oxidative stress of vascular cells is involved in the pathogenesis of diabetes, atherosclerosis, and cardiovascular disease (Incalza et al. 2018; Sena et al. 2018). Therefore, oxidative stress-mediated cell death is an important therapeutic target (Yao et al. 2021).

Nuclear factor-E2-related factor 2 (Nrf2) is an endogenous antioxidant protein and transcription factor that is highly sensitive to oxidative stress. Nrf2 binds to the antioxidant response elements of antioxidant genes; therefore, activation of the Nrf2 pathway is a promising therapeutic target to restore redox balance by reducing ROS (Lum and Roebuck 2001; Ma 2013; Galán et al. 2014; Chen et al. 2015). Nrf2 is activated by mitogen-activated protein kinase signalling under oxidative stress conditions (Sun et al. 2009). Extracellular-regulated kinases are involved in the oxidative stress response and plays a key role in the survival of endothelial cells (Chang and Karin 2001). Additionally, the accumulation of ROS in endothelial cells causes ER stress leading to cell dysfunction and death (Burgos-Morón et al. 2019).

The endoplasmic reticulum (ER) is a membrane-bound organelle that maintains cellular homeostasis under normal physiological conditions and ensures proper cell functioning, including the manufacture of proteins, synthesis of lipids, and the regulation of calcium (Schwarz and Blower 2016). Dysfunction or perturbation of the ER can cause cell damage through ER stress, which is a critical factor in the pathogenesis of various human diseases, including neurodegenerative and cardiovascular diseases (Ren et al. 2021). Accumulating evidence suggests that a disturbance in normal ER function plays a dominant role in inducing vascular dysfunction and endothelial cell death (Lenna et al. 2014). High blood glucose levels, hyperlipidaemia, insulin resistance, and oxidative stress cause endothelial cell dysfunction, which promotes the pathogenesis of blood vessels by inducing ER stress (Burgos-Morón et al. 2019).

The ER regulates protein post-translational modifications that control appropriate protein folding. During this process, disulphide bonds form the tertiary and quaternary structures of the protein and N-linked oligosaccharides attach to the nascent chain. A failure of appropriate protein folding triggers the degradation process of misfolded proteins called ER-associated degradation (Hampton 2002). Changes in ER function and misfolded proteins lead to ER stress. The cell attempts to restore ER homeostasis by triggering the unfolded protein response (UPR) pathway (Khanna et al. 2021). The UPR is a mechanism to remove unfolded/misfolded proteins and is a defense mechanism that prevents their accumulation. Two main degradation systems are involved in this defense mechanism, including proteasomes and autophagy. Cells determine the mechanism according to the type, severity, and duration of the stress (Ding et al. 2007).

**Figure 1.**
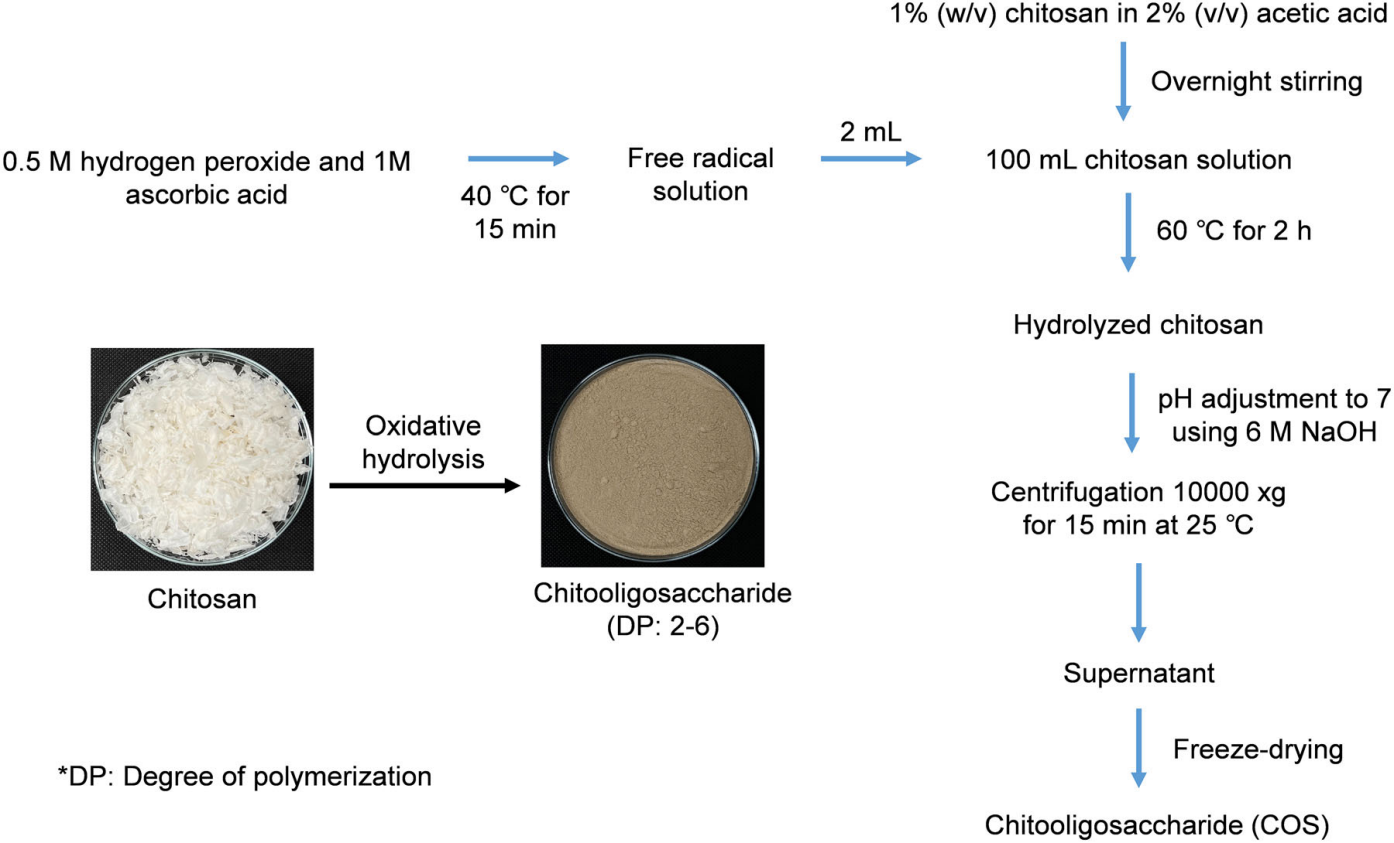
Preparation of chitooligosaccharides (COS) from the chitosan.

The production of chitooligosaccharides (COS) and chitin from acid digestion have been modified to obtain sufficient production and reproducibility for several applications (Figure 1). Recent research has suggested that COS are a potential pharmaceutically and biologically active substance (Lodhi et al. 2014). Because COS have considerably low toxicity with high water solubility, interest has grown to develop COS for medical applications. COS decrease cholesterol levels and blood pressure and possess radical scavenging, anti-inflammatory, and anticancer activities (Phil et al. 2018). COS scavenge free radicals and inhibit the NF-κB pathway after entering a cell (Mendis et al. 2007). Although the health benefits of COS have been widely revealed, information on the effect of COS on ER stress and their relevance to endothelial cells is limited. Therefore, the present study investigated the effect of COS on endothelial cell viability in response to ER stress and elucidated the underlying mechanisms.

## Materials and methods

### Preparation of chitooligosaccharide (COS)

Free radicals were generated by mixing 0.5 M hydrogen peroxide and 1 M ascorbic acid at 40 °C for 15 min as following the method of Fedoseeva et al. (2006) with slight modifications. Then, 1 g of shrimp shell chitosan (molecular weight: 1.5 × 10^6^ kDa) was dissolved in 2 mL/100 mL acetic acid overnight to get a final concentration of 1 g/100 mL. Next, 2 mL of radical solution was added into the 100 mL chitosan solution; the obtained mixture was incubated at 60 °C for 2 h with constant stirring using overhead stirrer equipped with a propeller (RW 20.n, IKA-Werke GmbH & CO.KG, Stau-fen, Germany). After the oxidative-reductive hydrolysis, hydrolysate was placed in the chilled water to cool down the mixture. The mixture was adjusted to pH 7 using 6 M NaOH followed by centrifugation using a centrifuge (Beckman Coulter, Inc., Palo Alto, CA) at 10,000 *g* for 15 min. The supernatant was collected and freeze-dried (CoolSafe 55, ScanLaf A/S, Lynge, Denmark) and the obtained powder was defined as ‘COS’. The degree of polymerization (DP) of COS was 2–8 as determined by matrix-assisted laser-desorption ionization time-of-flight (MALDI TOF) mass spectrometry (Tishchenko et al. 2011). The flow chart for preparation of COS was shown in Figure 1.

### Cell culture

The EA.hy926 cells, primary human umbilical vein cell line, were derived from the American Type Culture Collection (ATCC). The accession number of EA.hy926 from ATCC is CVCL_3901 (*Homo sapiens* (Human)). The EA.hy926 cells were cultured in Dulbecco’s Modified Eagle Medium (DMEM, high glucose medium, cat no: 12800-058) supplement with 10% foetal bovine serum (FBS), 1% of 100 units/mL penicillin/streptomycin and 1% of 2 mmol/L l-glutamine (Gibco, Gaithersburg, MA) at 37 °C in incubate with 5% CO_2_ until 70–80% confluence for further experiments.

### Cell viability assay

The EA.hy926 cells (0.8 × 10^4^ cells/well) were seeded into 96-well plates at 37 °C. The cells were treated with various concentrations of product of shrimp shell chitosan, COS (100, 250, 500, 1000 μg/mL). After 24 h incubation, MTT reagent (4 mg/mL) was dissolved in PBS and incubated for 3–4 h. After that, dimethyl sulphoxide (100 μL) was added to each well for dissolving the formazan crystals from mitochondria reduction and measure absorbance at 570 nm. Moreover, the cell viability was determined for ER stress inducer thapsigargin (Tg) (0.01–0.98 μg/mL) and tunicamycin (Tu) (0.025–8.17 μg/mL) on EA.hy926 cells.

### Cytoprotective activity for ER stress inducer-induced toxicity

The cytoprotective activity of shrimp shell chitosan was assessed on EA.hy926 cells by MTT assay. EA.hy926 cells were seeded at 0.8 × 10^4^ cells/well in 96 well plates. In the following days, pre-treated with COS (250, 500 μg/mL) for 24 h, subsequently ER stress inducer-Tg and Tu for toxic concentration of 0.16 μg/mL and 8.17 μg/mL, respectively, for 24 h. The protective effect of COS compounds against ER stress-induced EA.hy926 cells by MTT experiments.

### Nuclear staining assay

Apoptosis and necrotic cells death were evaluated by co-staining with Hoechst 33342 and PI. The EA.hy926 cells (0.8 × 10^4^ cells/well) were seeded in 96 well-plate and incubated at 37 °C. The cells were pre-treated with COS (250, 500 μg/mL) for 24 h, and then treated with ER stress inducers – Tg and Tu for toxic concentration for another 24 h. The treated cells were washed with PBS and co-stained with Hoechst 33342 (10 μg/mL) and propidium iodide (PI) (0.02 μg/mL) for 30 min. The fragmented nuclei for apoptosis cells stained with Hoechst 33342 and positive PI for necrotic cells were visualized by fluorescence microscopy (Olympus IX51 with a DP70 digital camera, Tokyo, Japan).

### Apoptosis assay by annexin V staining

Fluorescein isothiocyanate (FITC)-annexin V/PI apoptosis detection kit was used to determine early/late phase apoptosis and necrotic cell death, respectively, according to the manufacturer’s instruction (ImmunoTools, cat no: 3149001, Germany). The cells were incubated with Annexin V-FITC reagent and then PI in binding buffer for 15 min at room temperature. The stained cells were measured by flow cytometry (Benchtop Guava^®^easy Cyte HT system running with guavaSoft^TM^ software, version 3.3, EMD Millipore, Billerica, MA).

### Measurement of reactive oxygen species (ROS)

The intracellular ROS was determined by a fluorescent probe, DCFH_2_-DA (Sigma, St. Louis, MO). The cells were pre-treated with various concentrations of COS (250, 500 μg/mL) for 24 h followed by treatment with Tg 0.16 μg/mL for a further 24 h. Subsequently, the treated cells were probed with 10 μM DCFH_2_-DA for 30 min at a cool place. The fluorescence intensity was detected by a fluorescence microscope (Olympus IX 51 with DP70, Olympus America Inc., Centre Valley, PA). The measurement of superoxide anion level in treated cells was probed with 10 μM DHE. For the DHE assay, the protocol is the same as the DCFH_2_-DA probe.

### Real time quantitative PCR

The total RNA from treated cells (1 × 10^5^ cells) was extracted with GENEzol reagent. cDNA was synthesized from 1 μg of total RNA using SuperScript III reverse transcriptase (Invitrogen). RT-qPCR was performed in 100 ng of cDNA using Luna^®^Universal qPCR Master Mix (NEB, UK) with a final volume 20 μL and carried out in CFX 96 Real-time PCR system (Bio-Rad, Hercules, CA). The real time-PCR experiments with the following conditions: the initial denaturation step at 95 °C for 1 min, followed by 45 cycles of denaturation at 95 °C for 15 sec and primers annealing at 60 °C for 30 sec. The melting curve analysis was performed for primer specificity. The specific sequences of primers (Macrogen, Korea) were GRP78 forward (5′-GTTCTTCAATGGCAAGGAACCATC-3′), GRP78 reverse (5′-CCATCCTTTCGATTTCTTCAGGTG-3′), CHOP forward (5′-AGTGCCACGGAGAAAGCTAA-3′), CHOP reverse (5′-CCATACAGCAGCCTGAGTGA-3′), GAPDH forward (5′-CCACCCATGGCAAATTCCATGGCA-3′), GAPDH reverse (5′-TCTAGACGGCAGGTCAGGTCCACC-3′). For Nrf2, SOD1 and CAT gene were product of QuantiNova LNA Assay kit for product name HS_NFE2L2_1304872 (Cat no: SBH0061088-200), HS_SOD1_1522293 (Cat no: SBH0278498-200) and HS_CAT_2474653 (Cat no: SBH1219829-200), respectively. The relative mRNA expression levels of the targeted gene were calculated from the comparative Cq values. The PCR products were normalized with GAPDH genes as an internal control.

### Western blot analysis

EA.hy926 cells were treated with various COS concentrations (250, 500 μg/mL) for 24 h and removed medium. And then followed by treatment with Tg 0.16 μg/mL for a further 24 h. After that, the cells were harvested and washed with ice-cold PBS, then lysed with lysis buffer (50 mM 4-(2-hydroxyethyl)-1-piperazineethanesulfonic acid, pH 7.5, 150 mM NaCl, 5 mM EDTA, 1% Triton X-100, 1 mM phenylmethylsulphonylfluoride, 2 µg/mL pepstatin A, cat no: #9803, cell signalling) with complete protease inhibitor cocktail tablets provided in EASYpack (Roche, cat no: 04693116001) for 30 min, and centrifuged at 12,000 *g* at 4 °C for 15 min. The supernatant was collected and protein content was determined by using the Bicinchoninic acid (BCA) protein kit (Thermo-Fisher Scientific, Rockford, IL). The proteins were loaded on sodium dodecyl sulphate polyacrylamide gel electrophoresis (SDS-PAGE). Then, the proteins were transferred onto 0.45 µm nitrocellulose membrane by using semi-dry transfer method. The membrane was blocked with 5% non-fat milk in TBST (25 mM Tris-HCl (pH 7.5), 125 mM NaCl, 0.05% Tween 20) for 1 h and incubated with primary antibodies GRP78, PERK, Elf2α, p-elf2α, CHOP, PARP, ERK, p-ERK (Thr 202/Tyr 204), Nrf2, SOD1 (Cell Signaling Technology, Boston, MA) for overnight at 4 °C. β-Actin and GAPDH were used as loading control. All primary antibodies were prepared in 1:1000 in 5% w/v BSA in TBST. The following day, the membranes were incubated with horseradish peroxidase (HRP) conjugated with secondary antibodies IgG rabbit or mouse (Cell Signaling Technology, Boston, MA) for 1 h and complex reactivity was detected with chemiluminescence substrate.

### Immunofluorescence

EA.hy926 cells were incubated for cell concentration of 0.8 × 10^4^ cells/well in 96 well plates. In the following day, pre-treated with COS (250, 500 μg/mL) for 24 h, and then removed medium. After that, subsequently ER stress inducer-Tg (0.16 μg/mL) for toxic concentration for 24 h. After that, the cells were fixed with 4% paraformaldehyde for 15 min and permeabilized with 0.1% triton-X in FBS for 1 h. The cells were incubated with 1:400 of anti-GRP78 and CHOP for overnight at 4 °C. The cells were wash with 10% FBS in 0.1% Triton X and subsequently incubated with secondary antibody Alexa Fluor 488 (Invitrogen) conjugated goat anti-rabbit IgG (H + L), Alexa Fluor 488 (Invitrogen) conjugated goat anti-mouse IgG (H + L), and stained for nucleus with Hoechst33342 for 1 h at room temperature. The cells were washed with PBS and covered the cells with 50% glycerol. The immunofluorescence images were obtained by fluorescence microscope (Olympus IX 51 with DP70, Olympus America Inc., Center valley, PA).

### Statistical analysis

The data were presented as the mean ± standard deviation (SD) form three independent experiments. Multiple comparisons were done by one-way ANOVA analysis with post hoc test in Graph Pad Prism software (GraphPad Software, La Jolla, CA). The significant difference between groups was evaluated with a *p*-value < 0.05.

## Results

### Effects of COS and ER stress inducers on human EA.hy926 endothelial cells

**Figure 2.**
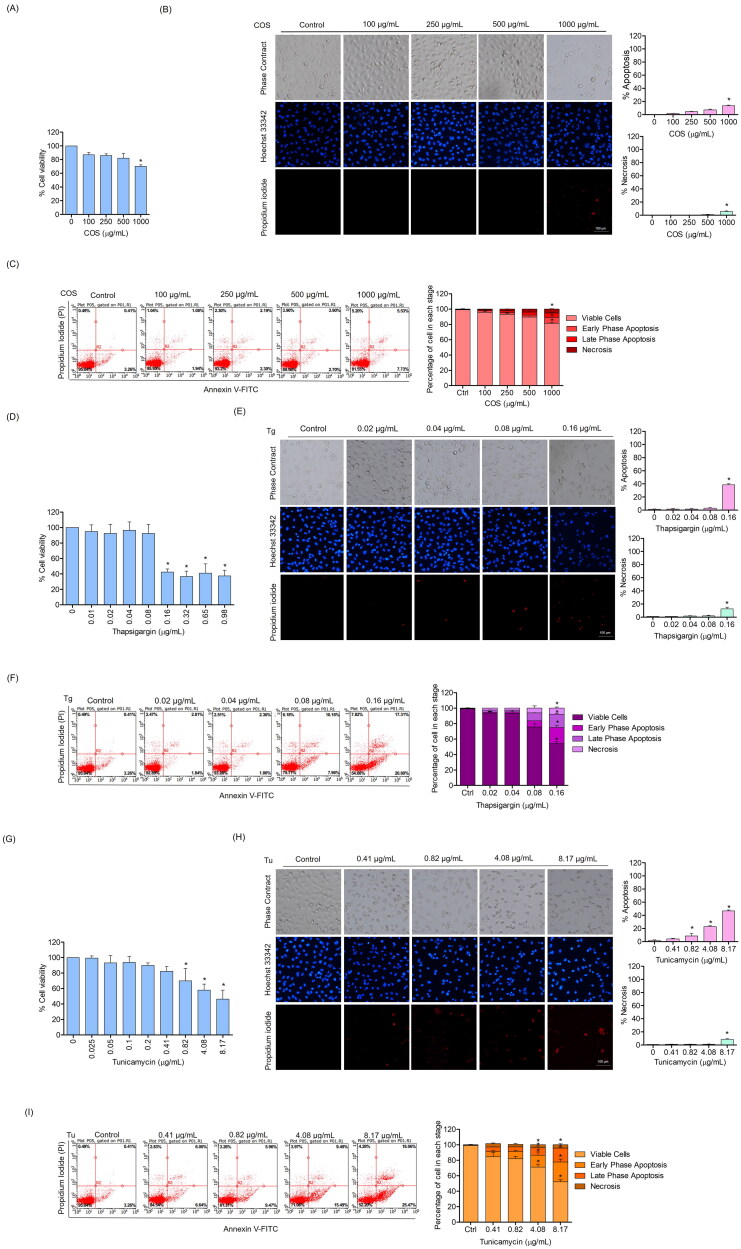
Cytotoxicity of COS and the ER stress inducers thapsigargin (Tg) and tunicamycin (Tu) on human endothelial cells. (A,D,G) Effect of COS and the ER stress inducers on the viability of EA.hy926 cells after 24 h as evaluated by the MTT assays; (B, E, H) morphology of apoptotic and necrotic nuclei evaluated by Hoechst33342 and propidium iodide (PI) co-staining and visualized using fluorescence microscopy; the percentages of apoptotic and necrotic cells were calculated; (C, F, I) Annexin V/PI dual staining of COS and the ER stress inducers added to EA.hy926 cells. Data are presented as mean ± SD (*n* = 3). Significant compared to the control group, *p < 0.05 versus un-treated control.

To verify the potential protective effects of COS on endothelial cell damage, the cytotoxic profiles of COS, and the concentrations of ER stress inducers required for the following experiments were determined. The cells were treated with various concentrations of COS (0–1,000 µg/mL) for 24 h, and cell viability was determined by the MTT assay. The results revealed that COS, at concentrations up to 500 µg/mL, caused minimal effects on cell survival (Figure 2(A)). The cells were treated under similar conditions and apoptosis and necrosis were determined by the Hoechst33342/propidium iodide (PI) nuclear staining assay. The apoptotic cells exhibited condensed or fragmented nuclei on Hoechst33342 staining, while the PI-positive cells were necrotic (Figure 2(B)). To confirm the apoptosis inducing effect of COS, the numbers of apoptotic and necrotic cells were investigated using Annexin V-FITC/PI staining. The flow cytometry results showed that Annexin V-FITC-positive cells (early phase apoptosis) and Annexin V-FITC/PI-positive cells (late phase apoptosis) appeared in cells treated with 1000 µg/mL of COS (Figure 2(C)). The cells treated with COS did not undergo apoptosis or necrosis at the indicated concentrations, suggesting that such concentrations were suitable for determining the protective effect of COS on endothelial cells.

To obtain the appropriate conditions for ER stress-induced endothelial cell damage, the toxic concentrations of the ER stress inducers thapsigargin (Tg) and tunicamycin (Tu) were investigated. EA.hy926 cells were treated with various concentrations of Tg (0–0.98 µg/mL) or Tu (0–8.17 µg/mL) for 24 h, and cell viability was measured. The results showed that 0.16 µg/mL Tg and 8.17 µg/mL Tu decreased cell viability to 42.36% and 46.21%, respectively (Figure 2(D,G)). The percentages of apoptotic and necrotic cells in response to ER stress induction were also determined. The results showed that Tg at concentrations up to 0.08 µg/mL had no apoptosis-inducing effect on these cells, while 0.16 µg/mL Tg induced 38.67% apoptotic and 12.5% necrotic cells (Figure 2(E)). The Annexin-V/PI assay by flow cytometry revealed that a Tg dose of 0.16 µg/mL caused 20.80 ± 2.7% early apoptosis, 17.31 ± 1.04% late apoptosis, and 7.82 ± 1.57% necrosis (Figure 2(F)).

Tu at concentrations of 0.41, 0.82, 4.08, and 8.17 µg/mL activated apoptotic cell death in 4.07 ± 1.1%, 8.67 ± 1.79%, 22.88 ± 1.53%, and 46.67 ± 1.53% of the cells, respectively. Notably, 8.71 ± 1.28% necrosis was detected in the cells that received 8.17 µg/mL Tu (Figure 2(H)). The early apoptosis, late phase apoptosis, and necrosis rates in response to 8.17 µg/mL Tu in the Annexin-V/PI assay were 25.47 ± 3.92%, 18.06 ± 0.55%, and 4.28 ± 1.29%, respectively (Figure 2(I)).

### Protective effect of COS on ER stress-induced endothelial cell death

**Figure 3.**
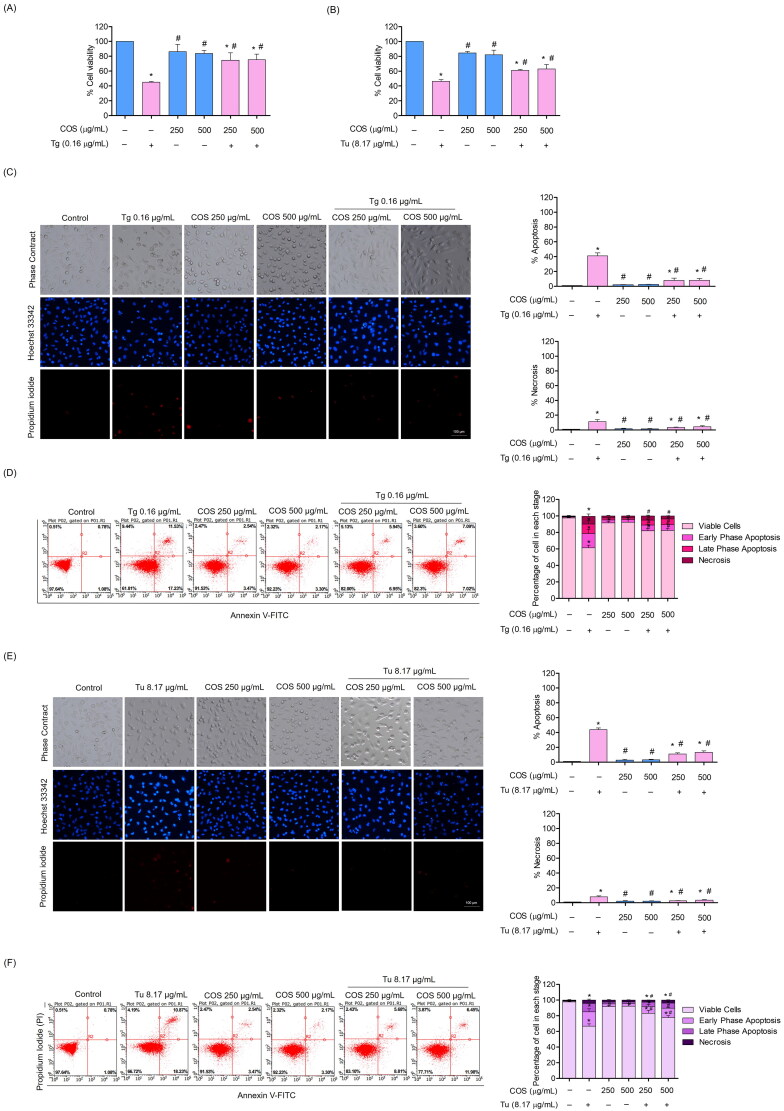
Cytoprotective effect of COS against ER stress in EA.hy926 cells. (A, B) The cytoprotective effect of COS on EA.hy926 cells against the toxicity induced by the ER stress inducers was investigated by the MTT assay; (C, E) morphology of apoptotic nuclei stained with Hoechst33342 dye and propidium iodide in cells treated with COS and the ER stress inducers (Tg and Tu). The results were visualized by fluorescence microscopy and the percentages of apoptotic and PI-positive cells were calculated; (D, F) flow cytometry analysis for Annexin V/PI staining after the COS pre-treatment (24 h) and treatment with the ER stress inducers for 24 h. Data are presented as mean ± SD (*n* = 3). *p < 0.05 versus control group and ^#^*p* < 0.05 versus Tg-treated group.

To evaluate the cytoprotective effect of COS against ER stress-induced apoptosis, the cells were pre-treated with COS at a non-toxic concentration followed by treatment with the ER stress inducers at toxic concentrations. Cells pre-treated with COS (250 or 500 µg/mL) for 24 h before Tg (0.16 µg/mL) was added were significantly protected against the toxicity caused by Tg. The cells viabilities of the COS-Tg treatment were 74.74 ± 3.95% and 75.34 ± 2.4% at 250 and 500 µg/mL, respectively compared with 44.97 ± 1% viability of the Tg treatment alone (Figure 3(A)).

To ensure the ER stress-protective effect, another ER stress inducer, Tu, was used at a toxic concentration (8.17 µg/mL). The results consistently revealed that pre-treatment of the cells with COS at the indicated concentrations increased cell survival in response to the Tu treatment. The cells viabilities of the COS-Tu treatment were 61.21 ± 1.11% and 63.06 ± 2.8% at 250 and 500 µg/mL, respectively, compared with 46.43 ± 2.06% viability of the Tu treatment alone (Figure 3(B)).

The percentages of apoptotic and necrotic cells in response to ER stress were also determined by the Hoechst33342 and PI co-staining assay. The results showed that the COS pre-treatment prevented apoptosis in EA.hy926 cells induced by the ER stress inducers. Treating the cells with 0.16 µg/mL Tg resulted in 41 ± 2.35% apoptosis. Cells pre-treated with 250 and 500 µg/mL COS before exposure to Tg revealed significantly reduced percentages of apoptosis of 8 ± 2.1% and 8.16 ± 2.56%, respectively (Figure 3(C)). The COS pre-treatment reduced apoptosis from 43.67 ± 2.6% (Tu treatment alone) to 11 ± 1.5% (250 µg/mL COS pre-treatment) and 13.17 ± 2.02% (500 µg/mL COS pre-treatment) (Figure 3(E)).

Additionally, the cells were pre-treated with COS, and apoptosis was determined by Annexin V-FITC/PI co-staining. The results revealed that cells treated with Tg alone exhibited higher apoptosis rates than that in the COS-Tu co-treated group. The early and late phase apoptosis rates in the Tg treatment alone were 17.23 ± 5.27% and 11.53 ± 4%, respectively. The 250 µg/mL COS pre-treatment before the Tu treatment caused early and late phase apoptosis rates of 6.9 ± 1% and 5.93 ± 1.3%, respectively. The 500 µg/mL COS pre-treatment caused early and late apoptosis rates of 6.95 ± 1.03% and 5.94 ± 1.2%, respectively (Figure 3(D)). Moreover, these results showed a similar effect of COS in protecting cells from ER stress using the ER stress inducer Tu (Figure 3(F)).

### COS suppresses the intracellular ROS production induced by thapsigargin

**Figure 4.**
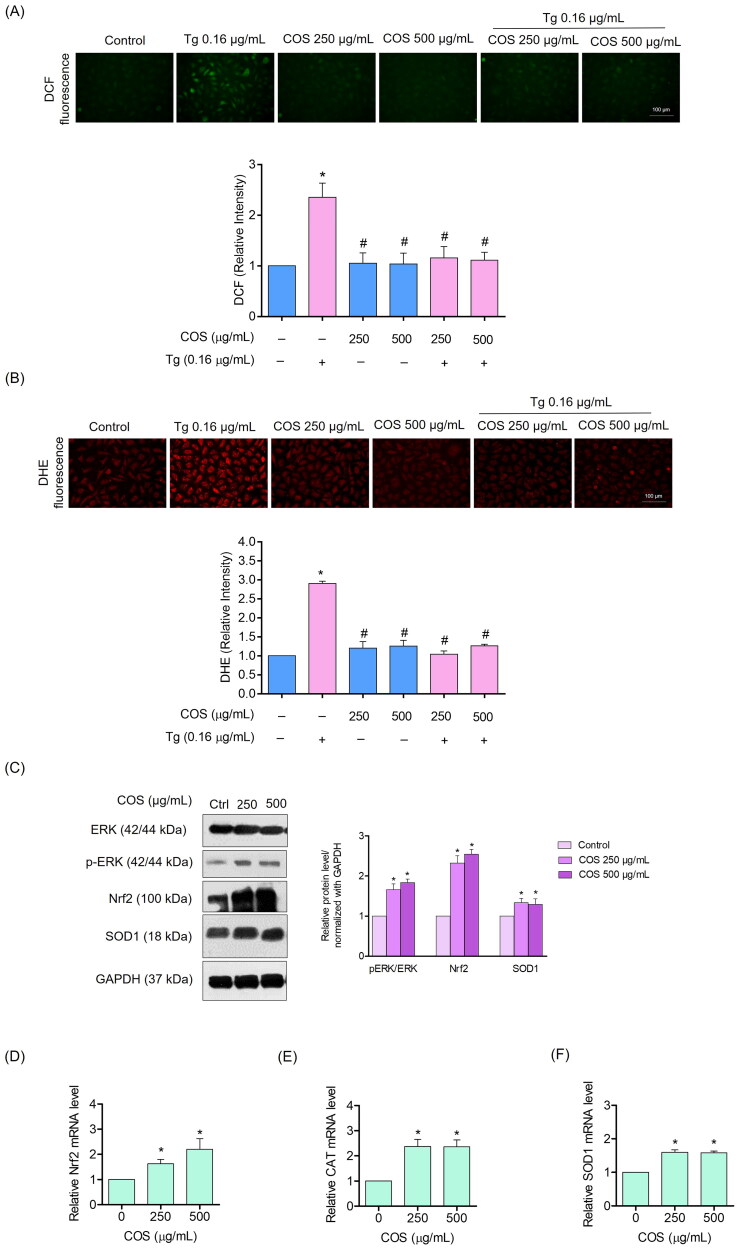
COS suppresses Tg-induced generation of reactive oxygen species (ROS) in EA.hy926 cells. The cells were pre-treated with COS for 24 h followed by the ER stress inducer Tg and were then incubated with DCFH_2_-DA or DHE for 1 h. The ROS levels were detected by (A, B) fluorescence microscopy. Effect of COS on ERK, Nrf2 antioxidant regulatory proteins, and antioxidants enzymes. The cells were treated with COS (250 or 500 μg/mL) and the antioxidant proteins and enzyme levels were determined. (C) ERK-pERK-Nrf2 and SOD1 proteins were determined by western blot analysis and the relative protein level was quantified by Image-J software (NIH Image J system, Bethesda, MD). GAPDH was used as the loading control; (D, E, F) mRNA levels for the Nrf2, catalase, and SOD1 genes were determined by quantitative real-time PCR. The data are shown as fold-change in mRNA expression normalized to GAPDH; data are presented as mean ± SD (*n* = 3). Significant compared to the control group, *p < 0.05 versus untreated control and ^#^*p* < 0.05 versus Tg-treated group.

Tg has been reported to induce cell death via an oxidative stress-dependent mechanism (Chaudhari et al. 2014) and ER stress is mediated by a ROS-dependent mechanism. The intracellular ROS levels in EA.hy926 cells were evaluated using a DCFH_2_-DA probe. The cells were pre-treated with COS (250 or 500 µg/mL) for 24 h and then treated with Tg (0.16 µg/mL) for 24 h. Fluorescence intensity was assessed with a fluorescence microscope. As results, pretreating the cells with COS significantly reduced the level of ROS induced by Tg, as shown in Figure 4(A), while Tg alone increased the ROS level compared to the untreated control cells.

Additionally, superoxide anions (O_2˚_^−^) were detected in the cells with the fluorogenic dye, dihydroethidium, which is a DHE probe. The treated cells were incubated with the probe, and DHE fluorescence intensity was determined. The results showed that the COS pre-treatment reduced the superoxide anions (O_2˚_^−^) generated by Tg (Figure 4(B)). Taken together, these results indicate that COS inhibits Tg-induced ROS generation in EA.hy926 cells.

### COS up-regulates Nrf2 and antioxidant enzymes in EA.hy 926 cells

Nrf2 plays an important role in regulating cellular antioxidant mechanisms. To investigate the COS inhibitory mechanism of ROS and superoxide anions induced by Tg, we determined the levels of Nrf2 and related antioxidant enzymes in COS-treated endothelial cells. The cells were treated with COS (250 or 500 µg/mL), ERK, p-ERK, and Nrf2 as well as the antioxidative enzymes, superoxide dismutase1 (SOD1) and catalase (CAT) were evaluated by western blot analysis. The results showed that the COS treatment significantly increased the Nrf2 level, which, in turn, induced the expression of the antioxidative enzymes. As the ERK signal has been shown to regulate Nrf2, we further demonstrated that COS increased the pERK/ERK level (Figure 4(C)). To confirm this result, the effect of COS on the mRNA expression levels of Nrf2, SOD1, and CAT were evaluated by real-time-polymerase chain reaction (RT-PCR) analysis. COS significantly increased the mRNA level of Nrf2, CAT, and SOD1 in endothelial cells (Figure 4(D–F)). These results suggest that COS activates ERK/Nrf2 antioxidant signalling.

### COS prevents ER stress-mediated cell death in endothelial cells by reducing the ER stress response

We have shown that COS suppressed ROS induced by Tg in endothelial cells and that ROS was associated with ER stress; thus, we next evaluated the possible link between COS suppressed ROS and Tg-mediated ER stress and cell death. Glucose-regulated protein 78 (GRP78) protein is a well-known regulator of the UPR and helps protect against the cell damage caused by ER stress by enhancing the protein folding process (Walter and Ron 2011). GRP78 is a known marker of ER stress (Zhu and Lee 2015).

**Figure 5.**
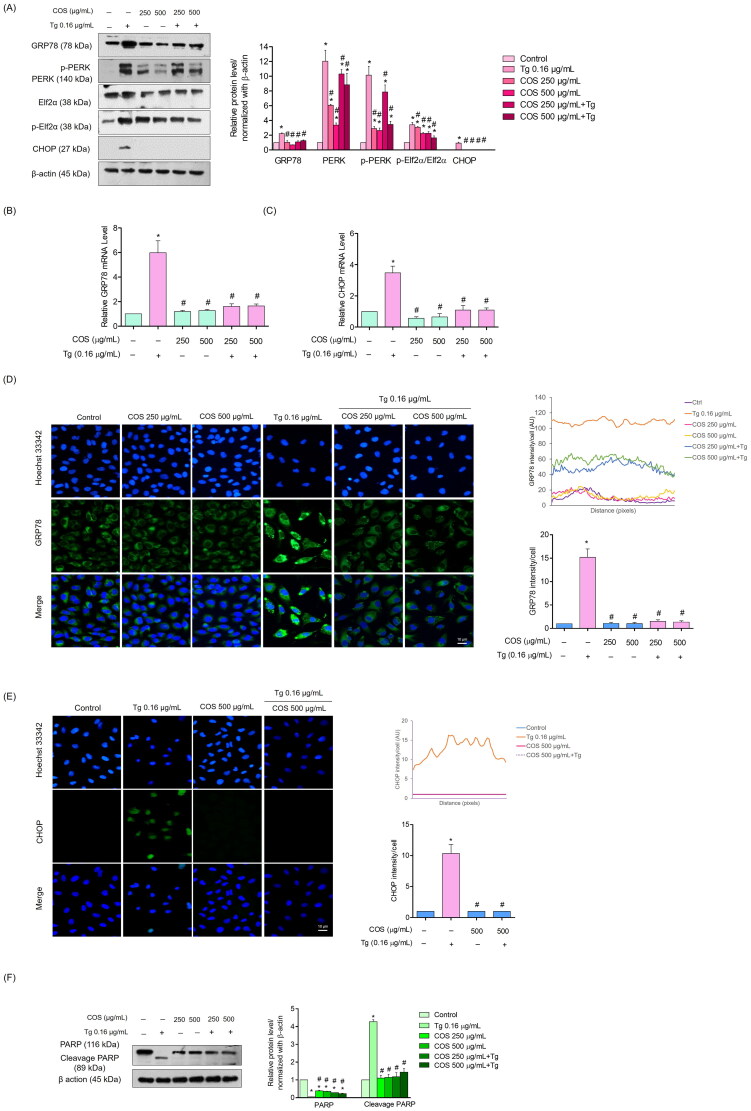
Characterization of the unfolded protein response to COS and the ER stress inducers. (A) The effect of the COS pre-treatment on the GRP78 protein level and the PERK-CHOP pathway was determined by western blot analysis; relative protein levels were calculated by densitometry. β-actin was used to confirm equal loading of the samples; (B, C) mRNA expression levels of GRP78 and CHOP were determined by quantitative real-time PCR. The data are presented as fold-change of mRNA expression normalized to GAPDH; (D, E) the GRP78 and CHOP proteins were detected by immunofluorescence. Fluorescence intensity was analyzed by Image-J software (NIH Image J system, Bethesda, MD); (F) the level of apoptosis-related protein PARP and its cleavage form were determined. Blots were normalized with β-actin to confirm equal loading of samples; data are presented as mean ± SD (*n* = 3). *p < 0.05 versus control group and ^#^*p* < 0.05 versus Tg-treated group.

Cells were exposed to COS, Tg, or pre-treated with COS before the Tg treatment. The expressions levels of GRP78 were determined by western blot, RT-PCR, and immunofluorescence analyses (Figure 5(A, B, D)). The results revealed that Tg significantly increased the basal level of GRP78 by approximately 2.5-fold, whereas the COS pre-treatment of endothelial cells reduced the cellular level of GRP78 compared with the Tg treatment alone. Moreover, the GRP78 gene expression level was upregulated in the Tg treated compared to the COS pre-treated cells (Figure 5(B)).

To confirm the effect of COS on GRP78, immunofluorescence analysis was performed to detect GRP78 in COS-treated cells, in the presence or absence of Tg. The cells were treated with COS and/or Tg, and the GRP78 protein was detected by immunofluorescence. Figure 5(D) shows that the basal GRP78 signal in control cells decreased in response to the COS treatment. As expected, the ER stress inducer Tg significantly increased the GRP78 signal. Pretreating the cells with COS before Tg exposure significantly reduced the GRP78-positive signal in the cells compared with Tg alone.

### COS pre-treatment attenuates ER stress-mediated apoptosis induced by thapsigargin

To further determine the mechanism of COS in regulating ER stress and apoptosis, the protein kinase RNA-like endoplasmic reticulum kinase (PERK) protein level was determined. The PERK protein is an important regulator linking ER stress and cell death. Therefore, we investigated whether the COS pre-treatment would attenuate the transduction of ER stress to apoptosis. Cells were pre-treated with COS (250 or 500 μg/mL) for 24 h and then treated with Tg (0.16 µg/mL) or left untreated for 24 h. The results showed that treating the cells with Tg significantly increased the phosphorylated p-PERK level. Pretreating the cells with COS at both concentrations only slightly attenuated the effect of Tg on inducing p-PERK (Figure 5(A)). A decrease in p-elf2α was observed after the COS pre-treatment (250 and 500 μg/mL) for 24 h.

Interestingly, the COS pre-treatment (250 or 500 μg/mL) dramatically reduced the CCAAT-enhancer-binding protein homologous protein (CHOP) in Tg-induced endothelial cells compared with Tg alone. As Nrf2 has been shown to suppress CHOP expression (Kasai et al. 2019), we determined whether the CHOP mRNA level could be suppressed by COS. Figure 5(C) shows that COS significantly reduced the CHOP mRNA level, while treatment with Tg increased CHOP expression. Pretreating with COS before Tg prevented the Tg-induced increase in the CHOP (Figure 5(A)) and mRNA levels (Figure 5(C)).

CHOP expression was also determined by immunofluorescence. The results revealed a high CHOP level in response to the Tg treatment alone, and the COS pre-treatment suppressed CHOP expression (Figure 5(E)). Taken together, COS, at least in part, suppressed CHOP expression by up-regulating Nrf2 (Figure 4(C,D)).

To link the effect of COS and ER stress-mediated apoptosis, we further analyzed apoptosis indicators in the same experimental setting. The levels of poly-ADP ribose polymerase (PARP) and cleaved PARP were determined by western blot analysis. The results revealed that the cells treated with Tg had high levels of cleaved PARP, an indicator of apoptosis, while COS did not affect the cleavage of the protein. Interestingly, pretreating the Tg-treated cells with COS significantly protected against PARP cleavage, suggesting that the apoptosis initiated by ER stress was blocked by COS (Figure 5(F)).

## Discussion

Oxidative stress is involved in several vascular diseases, such as atherosclerosis, diabetes, neuronal disorders, and ischemia-reperfusion injury. Moreover, ROS can lead to endothelial cell injury and death (Lum and Roebuck 2001; Sena et al. 2018; Shi 2022). ER stress was linked to cellular oxidative status, the ER stress inducer Tg induced ROS production, and ROS-dependent ER stress was the cause of endothelial cell death (Chaudhari et al. 2014; Galán et al. 2014; Plácido et al. 2015; Zeeshan et al. 2016).

The ER is an essential cell organelle that functions with the nuclear envelope to mature proteins, balance calcium, and synthesize lipids (Bravo et al. 2013). The main action of the ER is to control the conformation of folding proteins during post-translational modification that occurs in the ER lumen (Braakman and Hebert 2013). ER stress-mediated defects in normal cell function and ER stress-induced apoptosis participate in the development and progression of several human diseases (Ozcan and Tabas 2012). Vascular endothelial cells are highly sensitive to ER stress-mediated damage (Battson et al. 2017). Endothelial cells form a monolayer on the inner surface of vessels that are directly affected by chemical or mechanical changes in the lumen environment. ER stress can cause shear stress in the lumen of blood vessel, which promotes inflammation of endothelial cells (Bailey et al. 2017). Moreover, it has been demonstrated that shear stress induces human endothelial cell apoptosis via an ER stress-dependent mechanism (Pan et al. 2017). Several blood changes, such as high blood glucose, induce ER stress in endothelial cells (Dong et al. 2017). Moreover, pathological conditions, inflammation, and ROS disrupt the function of endothelial cells and are linked with increased ER stress (Zhou et al. 2021). Vascular function and integrity can be impaired by ER stress; thus, reducing ROS production, protects these cells from the damage induced by ER stress and could serve as a potential preventive approach to cardiovascular diseases (Panth et al. 2016). Here, we demonstrated for the first time the novel activity of COS in protecting endothelial cells from ER stress-mediated death by decreasing the generation of ROS.

It has been predicted that approximately 25% of proteins fail to be appropriately folded when ER activity reaches the maximum level (Battson et al. 2017). The accumulation of misfolded proteins triggers the control system called the UPR (Rutkowski and Kaufman 2004). The UPR attempts to shift the ER condition back to normal via several pathways, including inositol-requiring ER-to-nucleus signalling protein 1 (IRE1), RNA-dependent protein kinase-like ER eukaryotic initiation factor-2α kinase (PERK), and activating transcription factor 6 (ATF6) (Zheng et al. 2019). Activating PERK via protein phosphorylation results in a reduction of unfolded proteins suppressing translation through phosphorylation of eukaryotic translation initiation factor 2 (eIF2α). Activation of eIF2α activates transcription factor-4 (ATF4), which increases the production of the proteins responsible for protein folding and degradation (Rutkowski and Kaufman 2003). Evidence shows that activating PERK triggers apoptosis via an ATF4-dependent mechanism (Demay et al. 2014). Activating PERK in colorectal cancer cells has been recently demonstrated to cause ER stress-related apoptosis (Wu et al. 2020; Lei et al. 2021). In the present study, treating the cells with COS before inducing ER stress prevented the loss in cell viability and apoptosis. Our results show that preventing ER stress-mediated apoptosis was linked with the attenuation of CHOP-dependent apoptosis. CHOP is controlled by the Nrf2 and PERK pathways (Cullinan et al. 2003; Kasai et al. 2019). Our results indicate that COS increased the cellular Nrf2 level by activating ERK (Figure 4(C)). In addition, PERK slightly reduced the induction of p-PERK caused by the Tg treatment (Figure 5(A)). These results suggest that COS prevents ER stress-mediated death by inhibiting CHOP and suppressing Nrf2 and PERK.

The ER chaperone GRP78 is a regulator of ER stress that plays several key roles (Ni and Lee 2007). By enhancing ER activity to assemble nascent polypeptides, GRP78 prevents the misfolding and aggregation of proteins. In addition, GRP78 shifts the balance of ER stress back to normal by targeting misfolded proteins for degradation via the proteasomal degradation pathway (Lee 2001; Hendershot 2004). Our study showed that treating cells with non-toxic concentrations of COS slightly decreased the GRP78 level compared to the control, and the COS pre-treatment significantly suppressed the increase in GRP78 in response to Tg (Figure 5(A, B, D)).

The underlying mechanism of endothelial cell dysfunction and damage is associated with ER stress and ROS. Identifying the bioactive substances that attenuating ER stress and acts as antioxidants is important for preventing and treating relevant diseases. In addition, ER stress has been recently shown to regulate cell metabolism, survival, and degeneration. We showed that COS upregulated the cellular antioxidant enzymes SOD1 and CAT by inducing Nrf2 (Figure 4(C–F)). Consistently, the increase in ROS induced by the ER stress inducer Tg was significantly suppressed by the COS pre-treatment. As ROS are involved in the induction or enhancement of ER stress in many cells (Zeeshan et al. 2016), the antioxidant inductive effect of COS may help attenuate ER stress in endothelial cells.

## Conclusions

**Figure 6.**
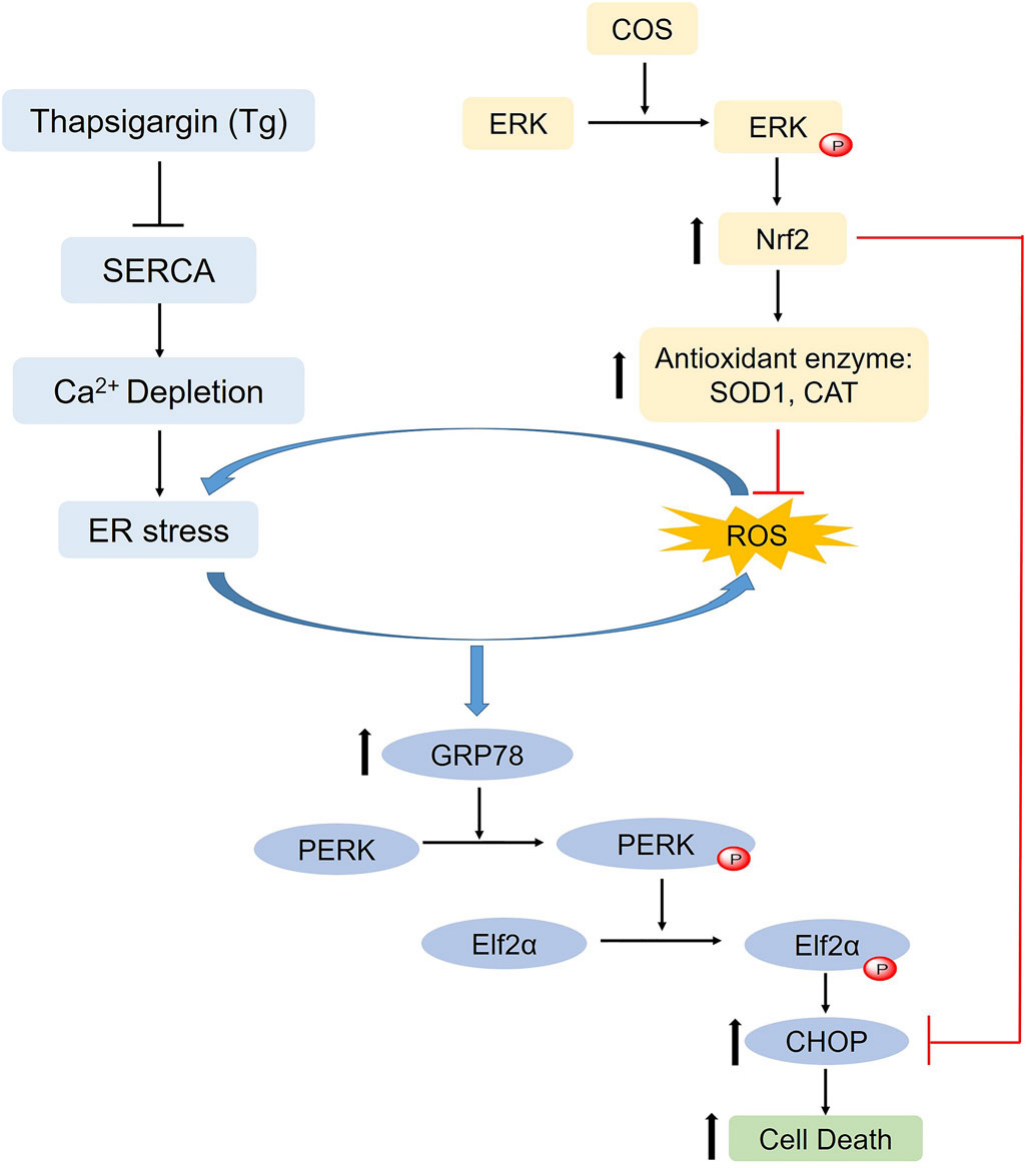
The underlying mechanism of COS in protecting Tg-induced ER stress and apoptosis of endothelial cells. Thapsigargin (Tg) is an inhibitor of sarcoplasmic reticulum Ca^2+^ ATPase (SERCA). Tg depletes calcium in the ER causing ER stress. ER stress increases intracellular ROS, and ROS triggers ER stress. ER stress-mediated cell apoptosis by the GRP78-dependent PERK-Elf2α-CHOP pathway. COS inhibits ROS generation by inducting Nrf2 and the antioxidant enzymes SOD1 and catalase. Moreover, COS activates ERK signals, leading to an increase in the Nrf2 level. Nrf2 is an inhibitor of CHOP expression. Overall, COS prevents ER stress-induced apoptosis by regulating oxidative stress, and the ERK-Nrf2-CHOP and PERK-Elf2α-CHOP pathways.

The present study revealed the novel activities of COS in protecting endothelial cells against ER stress-induced apoptosis by reducing ROS production via upregulation of Nrf2-dependent SOD1 and CAT and suppressing CHOP by increasing the cellular level of Nrf2 via ERK signalling (Figure 6). These results may help develop compounds and new ways to prevent disorders related to the death of endothelial cells and highlight the use of these relatively safe compounds for other health-benefiting applications.

## Sample availability

Samples of the compounds are available from the authors.
